# A time of transition: Stories from starting a new research program in 2020

**DOI:** 10.1016/j.isci.2021.102308

**Published:** 2021-04-07

**Authors:** Niankai Fu, Yingnan Hou, Kun Li, Mengying Li, Shuang Tang, Chao Yu

**Affiliations:** 1 Beijing National Laboratory for Molecular Sciences, Key Laboratory of Molecular Recognition and Function, Institute of Chemistry, Chinese Academy of Sciences, Beijing 100190, China; 2 School of Chemical Science, University of Chinese Academy of Sciences, Beijing 100049, China; 3 School of Agriculture and Biology, Shanghai Jiao Tong University, Shanghai 200240, China; 4 IDG/McGovern Institute for Brain research, School of Life Science, Tsinghua University, Beijing 100084, China; 5 Department of Mechanical Engineering, The Hong Kong Polytechnic University, Hung Hom, Kowloon, Hong Kong, China; 6 Cancer Institute and Department of Nuclear Medicine, Fudan University Shanghai Cancer Center, Shanghai 200032, China; 7 School of Environmental and Chemical Engineering, Jiangsu University of Science and Technology, Zhenjiang, Jiangsu Province 212003, China

**Figure:**
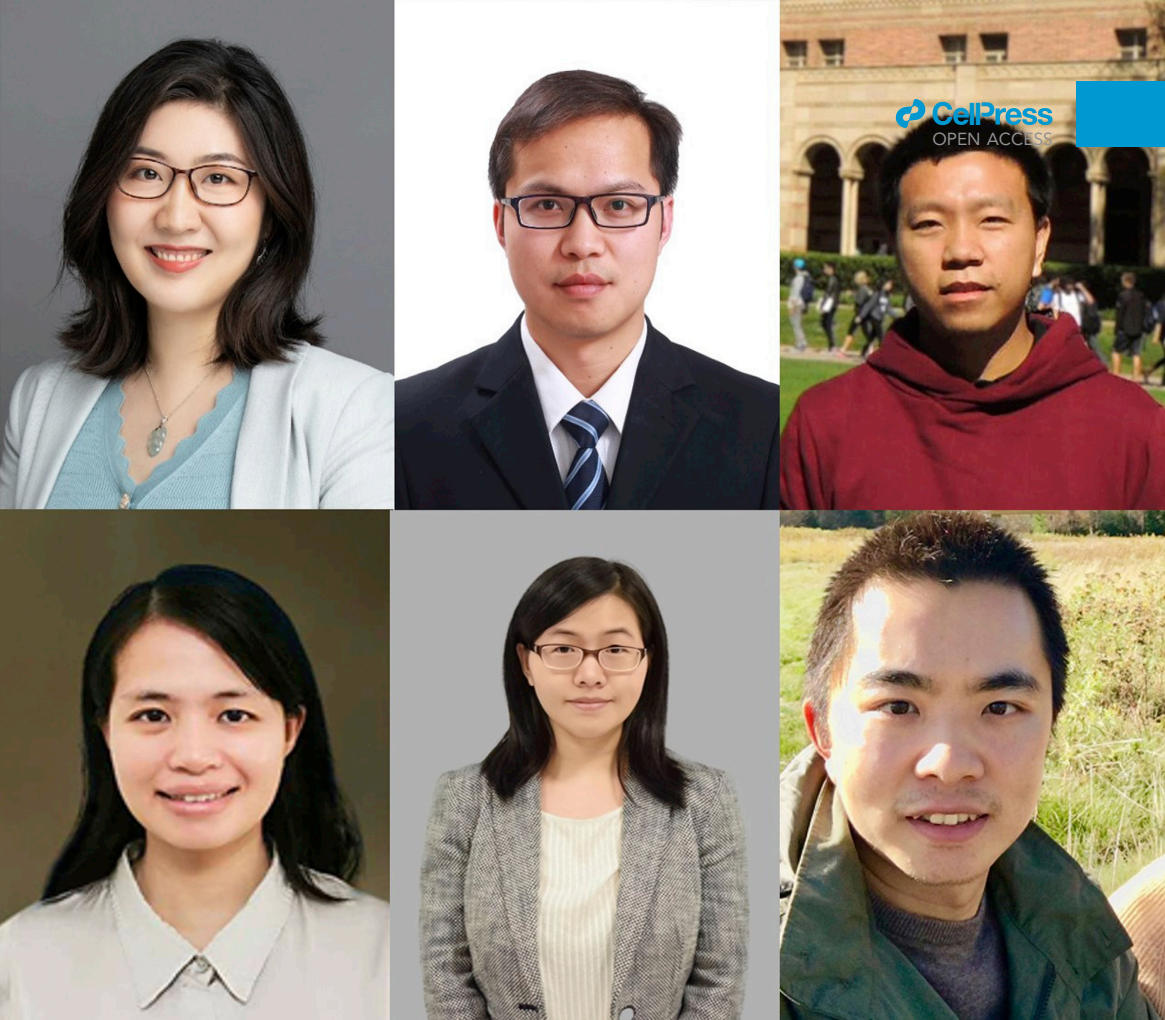
While everyone in the world in and out of academia has been affected by the pandemic, early-career researchers have had some of the most difficult challenges. Several professors who started and grew research programs in 2020 share their perspective and some advice to others in this Backstory. Top (from left): Mengying Li, Niankai Fu, and Yingnan Hou. Bottom (from left): Shuang Tang, Kun Li, and Chao Yu.

“The experience made starting a lab more precious and memorable.”

“Last but not least, I rebuilt my mind. I completed the transition from postdoc to principal investigator (PI), and so did my mind.”

“I asked for help from anyone who might be helpful. I received advice from my PhD supervisor, my postdoc supervisor, and other professors I knew.”

“We see the world around us changing, and we have to change accordingly.”

Starting a new research lab is one of the most stressful and exciting times in a scientist's life. Between becoming suddenly responsible for securing funding and lab space, the mentorship of trainees and, of course, teaching duties, entering the professorial ranks is, as an understatement, a time of transition. However, what if you were asked to take on all these responsibilities during or immediately after a global pandemic?

Here, we check in with six new faculty members who share their experiences in starting research labs in China and can reflect on each phase of the pandemic, from the early stages of confusion and anxiety to also the recovery and rebuilding. They have spent a long time mulling over the influence of the pandemic on their blossoming research programs, as well as reflecting on who has been pivotal to their journeys so far. From personal challenges that arise from the demands of flying across the world during a time of unpredictable travel bans to practical challenges that came from out-of-stock chemicals and supplies vital to research, these new professors have seen it all. Here, they document support of friends and colleagues in and out of academia, a strong reliance on family, and above all else, some hope for the future of science.

## Tough choices and beginnings

### How was the process of starting a lab in the past year?

**Mengying Li (The Hong Kong Polytechnic University):** I planned from early February and finally started the lab in late October, overcoming countless difficulties in between. The biggest difficulties were moving during a pandemic and the tough choice between work and family (whether to risk the kids' health and bring them to Hong Kong with me or move to Hong Kong by myself without seeing the kids for several months—I chose the latter with months of struggles). The experience made starting a lab more precious and memorable.

**Yingnan Hou (Shanghai Jiao Tong University):** I came back to China for several job interviews during the Christmas vacation of 2019. However, when I completed the trip for interviews, the pandemic began. With the shutdown of global travel, I started to stay at home and work remotely. Fortunately, I received an ideal offer from the university I'm working at. Without going back to the US, I accepted the new challenge directly. In the beginning, I had no idea how to set up a laboratory. Luckily, I got advice from experienced colleagues and had learned a lot by doing so. I worked hard in renovating the lab space, buying equipment, posting ads, and interviewing student and postdoc candidates. Now the lab has been running smoothly.

**Chao Yu (Jiangsu University of Science and Technology):** Its tough, especially this year. When the construction on my new lab started, I was still in the United States, waiting for the available flight back to China. At that time, my colleagues helped me immensely and installed the circuit, pipeline, drainer, and other basic facilities needed for my research work as we discussed and exchanged ideas online almost every day. When my 14-day quarantine and 14-day self-isolation ended, it was September. It's very lucky that there were two lecturers who joined my group and some of my friends in academia engaged while building the lab from scratch. Now the lab is beginning to take shape.

## Challenges

### What are any challenges you have faced in building your lab especially during a pandemic?

**Shuang Tang (Fudan University Shanghai Cancer Center):** This pandemic did significantly slow down the process for new faculty to start a lab. The time to setup the lab got prolonged, due to the slow down or lack of access to reagents and experimental supplies. Social distancing and isolation policies dramatically cut down the opportunity of communication which takes new faculty longer then to be familiar with the work environment and build a network. Moreover, collaborations between different institutions are difficult and complicated during the pandemic.

**Mengying:** The most challenging part for me was moving from the United States to Hong Kong during a pandemic with my whole family including a 2-month-old baby. Due to various travel restrictions and family obligations, I had to postpone the start of my lab by more than three months.

**Yingnan:** The biggest challenge is rebuilding—I needed to rebuild the lab space, which was designed originally as a lab for undergrads. Also, I had to rebuild my social relationships, especially within the academic society. I called all the people I knew in the academy and attended conferences in my field in the summer, just for reconnection. Last but not least, I rebuilt my mind. I completed the transition from postdoc to principal investigator (PI), and so did my mind. I started to learn how to communicate with my students and postdocs and how to lead a group forward to get the work done.

**Chao:** I found a lot of challenges with transportation and handling, and it was frustrating as we can adjust and accommodate to such challenges but are unable to fully control them. For example, we planned to build a catalytic unit according to our experimental plan. There was one heating apparatus needed to be purchased from a Korean vendor. However, the delivery date was postponed, leading to the sudden change of our plans. It takes a lot of energy to course-correct.

## Training and mentorship

### Was it hard to recruit students/postdocs into your lab as you started?

**Niankai Fu (Institute of Chemistry, Chinese Academy of Sciences):** I started my lab in Beijing in November 2019. After the Spring Festival, our institute was partly shut down. Thanks to the efficiency of our government and people have done in preventing the spread of the coronavirus, we were lucky to be able to work in the office in February. Soon after, we could do experiments in the lab, but chemicals and equipment deliveries were generally delayed due to the pandemic. In addition, it was difficult to recruit postdocs during the pandemic. Even in the case that I was satisfied through a virtual interview to get the candidate to work with me in the lab, he/she just could not come to work immediately because of the travel restrictions. In terms of students, the only graduate student I have was suggested to stay at home at the time, considering the risk of traveling across the country. To our delight, we are fully back in business since July and looking forward to the future.

**Kun Li (Tsinghua University):** Due to the spread of COVID-19 virus around the world, many countries have issued travel restrictions and suspended student visa application services. Most Chinese students who originally planned to study abroad decided to get Ph.D. or post-doctoral research training in China. It's a good time for young PIs in China to recruit top students graduated from the best Chinese universities, but new labs still have challenges in attracting postdocs compared to well-established labs.

**Mengying:** For me, I think the pandemic actually helped to recruit students. Due to the severe pandemic conditions in the United States and Europe, more students from mainland China chose Hong Kong as their first choice to have a different experience.

**Yingnan:** There is no “easy” way to start a lab, much less doing so in 2020. I posted my personal website and ads for recruiting just after onboarding. I also asked my friends in the academy to help repost my ads. One student and one postdoc candidate contacted me. I had online interviews with them due to travel restrictions. I have to say that I was kind of lucky. I found we matched very well with each other. We are working in the lab together now.

### Have you engaged in any interdisciplinary collaborations to start your lab? If so, were they inspired by you or your institution?

**Shuang:** Yes, my research focus on cell metabolism and nuclear medicine, which requires me to engage in broad collaborations between life science with clinical medicine, as well as medical engineering. The collaborations were highly inspired by my institution, especially for the internal collaborations.

**Chao**: Not yet. But we do have researchers from various fields in local areas which is something that has been on my mind now since I started the position. Talking is the first part to start a collaboration, so we are going to organize interdisciplinary seminars if the coronavirus allows us.

### Where did you get help from or seek advice from in establishing your lab during this time?

**Kun:** We have faculty lunch seminars and happy hour every week in our department, and the senior faculty will share with us their valuable experience in establishing and running labs.

**Mengying:** I got help and sought advice from my PhD advisor, some of my current colleagues, and friends who are in academia. Due to the pandemic, all the discussions and chats are online, which I think it is helpful in getting information but less effective in connecting with them, especially with new colleagues. For example, I was planning to invite one colleague per week to lunch to know more about their research, seek advices, and get familiar with the departmental culture, but the pandemic made the planning impossible.

**Niankai:** Of course, the senior colleagues at our institute. I earned my PhD at the same institute—I am quite familiar with all professors in the department, and they are really supportive. I will turn to them whenever I have questions about running the new lab. Also, my postdoc experience with Professor Song Lin at Cornell gave me some insight into what to expect when starting a lab, as I joined his lab when he was just starting his independent work.

**Yingnan**: I asked for help from anyone who might be helpful. I received advice from my PhD supervisor, my postdoc supervisor, and other professors I knew. They gave me the most important suggestions to be a good and successful PI. I also visited several experienced professors at my university. They shared with me detailed advice in establishing and running a lab at the university.

## Words of advice

### What is the most important thing you want to share with others?

**Shuang:** Be prepared to face uncertainty and plan everything early.

**Niankai:** We see the world around us changing, and we have to change accordingly. No one is perfect at this job, but try to be positive. We are chemists; we do experiments. It is fine to get some negative results, but we should be confident that we know how to optimize the conditions for better reaction outcomes.

**Yingnan:** I want to say three important things: BE ACTIVE! BE ACTIVE! And BE ACTIVE! Send an email, make a call, and talk with someone in the academy or at your university every single day. Any suggestions from them could be very helpful for a fresh PI.

**Mengying:** I entered academia to start my own lab, had my second baby, and moved across the Pacific Ocean, all during the pandemic. I think the pandemic taught me to embrace uncertainties and adjust plans accordingly, identify what matters most, and be more focused.

**Chao:** My father didn't forget the birthday cake during my 14-day quarantine time. Family support always backs us up when there is a rainy day. I, even after I had left home for nearly five years, fit into this new situation and environment immediately amid the chaotic feelings of the pandemic, just because my parents and family are my rock here.

## About the authors' research programs

*Niankai Fu (Institute of Chemistry, Chinese Academy of Sciences):* Electrochemistry can convert even the most tenacious functionalities into highly reactive and enabling radical species under extremely mild reaction conditions in a sustainable fashion. Transition metal catalysis lies at the heart of modern synthetic chemistry as one of the most active and creative fields of research and has been continually demonstrating new modes of chemical activation for the discovery of unprecedented transformations. By bridging electrochemistry and transition metal catalysis, we develop novel electrocatalytic strategies to tackle daunting challenges in catalysis and synthetic science. We are now particularly interested in addressing long-standing problems in asymmetric catalysis by taking advantage of the intimate interactions of electrochemistry and transition metal catalysts with organic molecules that can be rationally designed for facile and selective conversion of readily available starting materials into value-added products.

*Yingnan Hou (Shanghai Jiao Tong University):* My lab focuses on the interactions between plant hosts and microbial pathogens. Crop losses caused by biotic stress are estimated to be between 20% and 40% every year. Filamentous eukaryotic pathogens, including fungi and oomycetes, are major threats to economically important crops. Most filamentous plant pathogens are biotrophic or hemibiotrophic, engaged in a continuous battle with plant hosts by exchanging active materials to manipulate each other. Oomycetes are known for secreting a large number of effectors to modulate host immune responses, while plant produces antimicrobial agents to defeat non-viral pathogens. Therefore, a deep understanding of the molecule exchanges between pathogens and hosts is crucial for elucidating virulence mechanisms. In my laboratory, I will mainly focus on understanding the complicated interplay between plants and pathogens.

*Kun Li (Tsinghua University):* My lab focuses on understanding the neural mechanisms responsible for sexual dimorphism in social and emotional behaviors. We take advantage of pathway-specific and activity-dependent translating ribosome affinity purification approaches, combined with other state-of-the-art techniques, including single nuclei sequencing, optogenetics, electrophysiology, and calcium imaging to identify sexually dimorphic modules in the brain. The goal of our lab is to elucidate how sex differences in the brain at the levels of molecules, cells, and circuits cause gender differences in behaviors.

*Mengying Li (The Hong Kong Polytechnic University):* My research focuses on the science and technologies for renewable energy utilizations, aiming to mitigate the abrupt climate change while sustaining the energy, food, and water supplies. I am particularly interested in renewable energy integration by developing physics-based, remote sensing, and machine learning-integrated adaptive technologies for solar energy resourcing and forecasting. I am also interested in the design of multi-generation renewable systems that co-produce power, heat, cooling power, hydrogen, food, and fresh water. For more info, please visit: www.li-realab.info.

*Shuang Tang (Fudan University Shanghai Cancer Center):* My research focuses on metabolism and nuclear medicine. Using isotope labeling methods together with multi-pronged approaches in cell biology, molecular biology, metabolomics, and mouse models, my team aims to understand how metabolism and protein modifications regulate the adaptation of fast-proliferating cells (stem cells and cancer cells) to microenvironment and translate the knowledge for precise monitoring and treatment of cancer by combining with metabolic molecular imaging.

*Chao Yu (Jiangsu University of Science and Technology):* My research interest is on developing new assembly methods of nanomaterials and their applications for greener and more sustainable heterogeneous catalysis. Starting from bottom-up controllable synthesis and micro-nano self-assembly technology, I have developed economic nanocatalysts and analyzed the regulation mechanism of the intermediate state/transition state/final product in various catalytic steps to realize the integration of multi-step process routes and hope to continue in this direction as establishing my lab.

**Figure:**
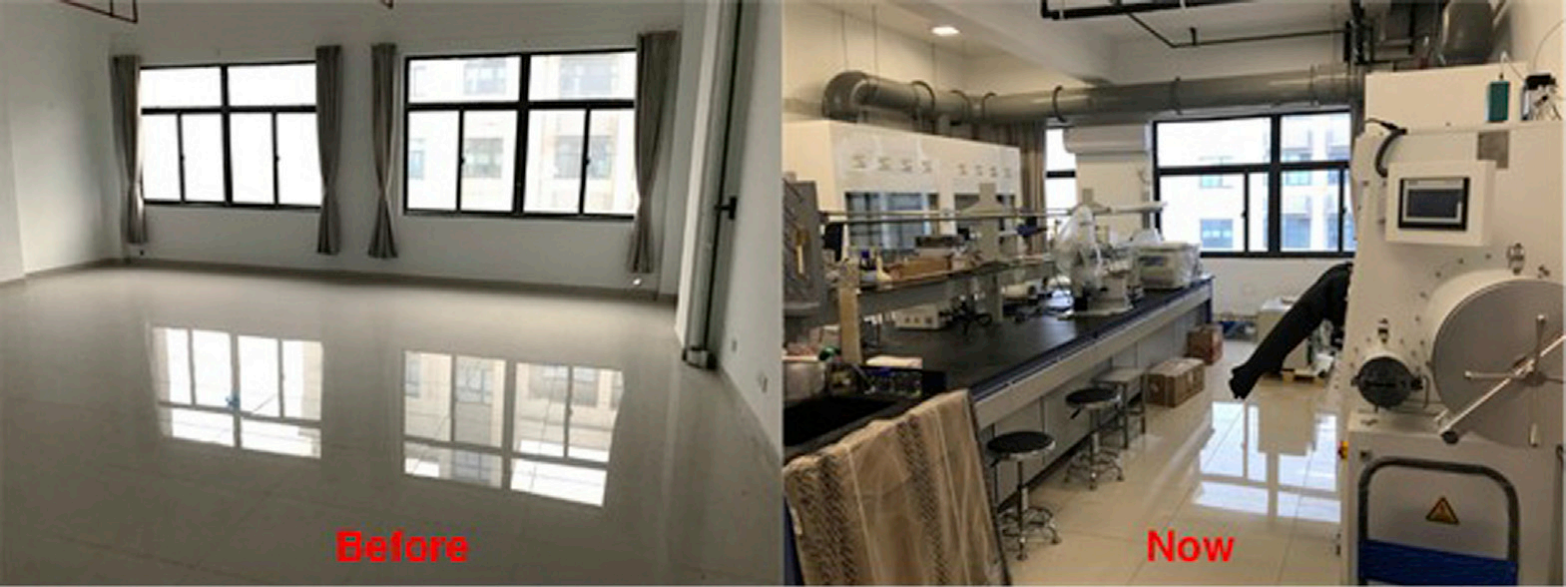
The pandemic led to uncontrollable product shortages and delays in shipments that presented a huge challenge for those starting a lab in the past year. However, in these images courtesy of Chao Yu, where there was once nothing, now there is a growing lab and research program.

**Figure:**
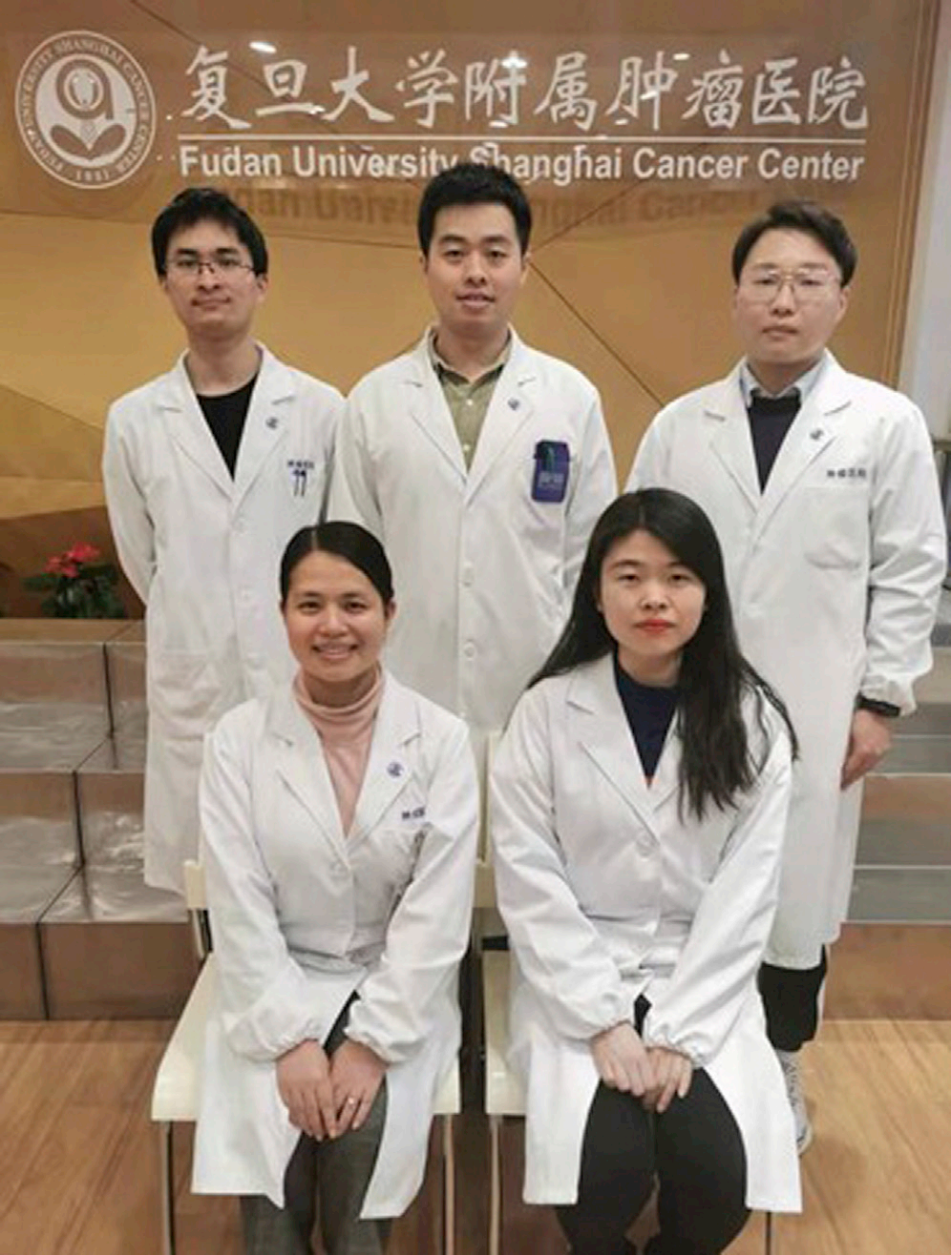
Recruiting students and postdocs was a clear challenge during the pandemic because of travel bans and reliance on virtual communication. In this photo courtesy of Shuang Tang, she is seen with her collaborators. First row (from left): Shuang Tang, Qian Zhou. Second row (from left): Wei Liu, Yi Liu, and Weijing He.

